# Growth of uncertainty: how fragmented abortion legislation associates with maternal healthcare engagement and outcomes- an ecological cross-sectional analysis of secondary state-level data

**DOI:** 10.1186/s12889-026-28998-3

**Published:** 2026-08-13

**Authors:** Mark Clunes, Gustavo Gabriel Huaman, Alexander Gazo, Yusuf Yakubu, Chadae Louison, Tonia Frame

**Affiliations:** 1 Medical School Research Institute, School of Medicine, St. George’s University, St. George’s, West Indies Grenada; 2 Department of Public Health and Preventive Medicine, School of Medicine, St. George’s University, St. George’s, West Indies Grenada; 3 Department of Physiology, Neuroscience and Behavioral Science, School of Medicine, St George’s University, St. George’s, West Indies Grenada

**Keywords:** Abortion legislation, Maternal health outcomes, Reproductive health policy, Perinatal health outcomes, United States

## Abstract

**Background:**

Following *Dobbs v. Jackson Women’s Health Organization*, abortion policy in the United States has become increasingly fragmented across states. This study examined whether legislative restrictiveness is associated with differences in maternal and perinatal health at the population level.

**Methods:**

This ecological cross-sectional study assessed state-level associations between abortion-policy restrictiveness and maternal age distribution, intended pregnancy, prenatal care timing and adequacy, gestational age, birth weight, infant mortality subcategories, insurance coverage, neonatal intensive care unit admission, and obstetric service access across the 50 states and the District of Columbia. Policy data from the Guttmacher Institute and the Center for Reproductive Rights were harmonized into a five-point restrictiveness scale. Health indicators were obtained primarily from March of Dimes PeriStats (2020–2025) and supplemented with national datasets. State values were standardized as Z-scores and analyzed using Spearman rank correlations, Kruskal–Wallis tests, and Bonferroni-adjusted post hoc comparisons.

**Results:**

Increasing legislative restrictiveness was statistically associated with lower intended pregnancy prevalence, reduced early prenatal care, higher rates of moderate preterm birth, and increased moderate and very low birth weight. More restrictive states also demonstrated younger maternal age distributions. Observed associations ranged from moderate to strong in magnitude.

**Conclusions:**

Although causal inference is limited by the ecological cross-sectional design, findings suggest that abortion-policy fragmentation is associated with broader patterns of reproductive health disadvantage in the post-*Dobbs* United States, with potential implications for maternal and perinatal outcomes.

## Background

The Supreme Court’s decision in Dobbs v. Jackson Women’s Health Organization, 597 U.S. 715 (2022) [1], overturned Roe v. Wade, 410 U.S. 113 (1973) [2], and ended the federal constitutional framework that had shaped abortion access for nearly half a century. Since then, abortion regulation has become increasingly state-dependent, with some jurisdictions protecting access in statute or state constitutions and others imposing total bans with limited exceptions or severe criminal and civil penalties [3].

This policy shift matters for more than abortion service volume alone. State policy fragmentation has altered where care is available, how far patients must travel, and the degree of legal certainty facing clinicians, patients, and health systems. Guttmacher Institute estimates indicate that nearly 170,000 abortion patients traveled across state lines for care in 2023 and 155,000 did so in 2024, compared with 81,000 in 2020, underscoring the rapid geographic redistribution of access in the post-Dobbs era [4, 5].

A growing literature suggests that restrictive abortion-policy environments are also associated with broader maternal and infant health risks. Ecological and quasi-experimental studies have linked more restrictive policy climates to higher maternal mortality, adverse obstetric outcomes, and higher infant mortality, even after accounting for secular trends and state-level covariates [6–14]. These studies do not imply that abortion policy is the sole cause of outcome differences, but they do suggest that reproductive legislation may function as both a direct constraint and a marker of wider structural conditions affecting maternal health.

Two complementary mechanisms are especially relevant to the present analysis. First, pregnancy intention is associated with prenatal care use and maternal-infant outcomes; unintended pregnancy has been linked to delayed or reduced antenatal care and several adverse health outcomes [15, 16]. Second, comprehensive sexuality education is associated with improved contraceptive knowledge and lower teen birth rates, suggesting that policy environments shaping reproductive autonomy may influence maternal age structure and pregnancy planning before pregnancy occurs [17, 18].

This study examines whether state-level abortion-policy restrictiveness is associated with measurable differences in maternal and neonatal health indicators across the United States. Using an ecological cross-sectional design, it integrates state policy measures with perinatal, prenatal care, insurance, and obstetric-access indicators from national public datasets for 2020–2025.

The working hypothesis is that states with more protective reproductive policy environments will exhibit higher intended pregnancy prevalence, earlier prenatal engagement, and more favorable gestational-age and birth-weight profiles, whereas more restrictive states will exhibit younger maternal age distributions, lower intended pregnancy, delayed prenatal care, and worse neonatal outcomes. Because the study is ecological, the analysis is intended to characterize population-level patterning rather than establish individual-level or causal effects.

## Methods

### Study design and abortion laws and policies data sources

This ecological cross-sectional analysis compared state-level maternal and neonatal health indicators across a harmonized five-point abortion-policy restrictiveness scale drawn from the Guttmacher Institute’s Interactive Map: U.S. Abortion Policies (https://states.guttmacher.org/policies/kansas/abortion-policies) and Policy data drawn from the Center for Reproductive Rights’ After Roe Fell: U.S. Abortion Laws by State (https://reproductiverights.org/maps/abortion-laws-by-state/?focus=29), both accessed October 1, 2025 [3, 4]. Please refer to the websites for geographic mapping of the legislative information.

### Data transformation and description

The two source frameworks were mapped onto a single five-category scale ranging from 1 (most protective) to 5 (most restrictive). For each state, the corresponding scores on the 7-point Guttmacher scale and the 5-point reproductive-rights scale were treated as paired observations and displayed in a scatter plot. A linear regression line was fitted to the paired scores. The relationship between the two scales was strong, with a Spearman’s correlation coefficient of (**ρ** = 0.92). The fitted relationship was then used to estimate the corresponding 5-point value for each category of the Guttmacher 7-point scale. Based on this analysis, scores of 1 and 2 on the 7-point scale mapped to 1 on the 5-point scale; 3 mapped to 2; 4 and 5 mapped to 3; 6 mapped to 4; and 7 mapped to 5. The average of the reproductive rights 5-point scale and the Guttmacher mapped 5-point scale were then taken and rounded to a whole integer for each state.

Qualitatively this numerical mapping also aligned; Guttmacher categories were collapsed where they represented narrower distinctions within the broader 7-point classification when mapped to the 5-point scale. Guttmacher scores 1–2, both indicating multiple state-level legal protections for abortion access, provision, and confidentiality, were mapped to category 1 on the 5-point scale, defined as reproductive rights protected by state law and policy. Guttmacher scores 4–5, in which abortion remained legally available, but access was increasingly limited by eligibility conditions, Medicaid exclusions, or insurance restrictions, were mapped to category 3, defined as accessible but not securely safeguarded by law. The remaining categories showed clearer one-to-one correspondence: Guttmacher score 3, indicating protection with some limitations, mapped to category 2, defined as protected but with limitations to access; score 6, indicating limited availability under restrictive and/or hostile conditions, mapped to category 4, defined as hostile with no legal protection and plans to expand restrictions; and score 7, indicating a complete ban and criminalization, mapped to category 5, defined as illegal and criminalized.

### Maternal and infant health data source

Outcome data were obtained primarily from the March of Dimes PeriStats database for 2020–2025 and supplemented with March of Dimes maternity care access measures, National Center for Health Statistics demographic data, and SIECUS state policy profiles for contextual comparison [19–22]. The 2025 SIECUS State Report Card assigns each state an overall grade based on two weighted domains: state requirements for sex education and requirements concerning curricular content under state law or mandated educational standards. The grade therefore characterizes the strength and comprehensiveness of the state’s sex-education policy framework rather than its implementation at the local level. For this analysis, the 13 possible grades (A+, A, A−, B+, B, B−, C+, C, C−, D+, D, D−, and F) were reverse-coded from A + = 1 to F = 13; higher values indicated a policy environment less supportive of comprehensive sex education [23].

Indicators included maternal age distribution (< 20, 20–29, 30–39, >=40 years), gestational-age distribution, birth-weight distribution, infant mortality subcategories, prenatal care timing and adequacy, counties without obstetric services, NICU admission, uninsured women, intended pregnancy, median household income, and a state sex education policy grade used descriptively as a contextual indicator. All 50 states and the District of Columbia were included when data were available. Missing observations were left blank without imputation. Gestational-age and birth-weight categories followed March of Dimes PeriStats definitions [19]. Preterm-birth was defined as birth before 37 completed weeks of gestation, moderately-preterm birth as 32 to less than 37 weeks, and very-preterm birth as less than 32 weeks; births at 37 weeks or later were classified as not-preterm. Low-birth weight was defined as less than 2,500 g, with moderately-low birth weight defined as 1,500–2,499 g and very-low birth weight as less than 1,500 g; births weighing at least 2,500 g were classified as not-low birth weight. Other PeriStats outcomes were defined according to the source documentation [19]. Early-prenatal care indicated initiation during the first trimester (months 1–3), second-trimester care indicated initiation during months 4–6, and late or no prenatal care indicated initiation during months 7–9 or no prenatal care. Prenatal-care adequacy was classified using the Adequacy of Prenatal Care Utilization Index, which combines the timing of initiation, the number of visits received, and the number expected for the infant’s gestational age. Adequate or adequate-plus care indicated initiation before the fifth month with at least 80% of the expected visits, intermediate-care indicated initiation before the fifth month with 50%–79% of the expected visits, and inadequate-care indicated initiation during or after the fifth month or receipt of fewer than 50% of the expected visits. Intended pregnancy was defined as the percentage of mothers reporting that they had wanted to become pregnant at that time or sooner. NICU admission represented the percentage of live-born infants admitted to a neonatal intensive care unit. Infant mortality was defined as death during the first year of life, and cause-specific infant mortality measures were expressed as deaths attributed to the reported cause per 100,000 live births.

### Data analysis

For each outcome, state-level values were standardized as Z-scores across the national distribution for that variable. This allowed comparison of relative state standing across indicators that were originally reported on different scales. Descriptive visualization used standardized heat maps, ranked plots, and grouped boxplots to display national gradients across the restrictiveness continuum.

Associations between legislative restrictiveness and state-level outcomes were assessed using Spearman rank correlation, given the non-linear, but monotonic relationships. Spearman rank correlation was used to assess monotonic relationships. Kruskal-Wallis tests were used to compare outcomes across the five restrictiveness categories. Where omnibus results were statistically significant, post hoc Mann-Whitney U tests with Bonferroni adjustment were conducted.

Analyses were performed in Python 3.11 using open-source packages including pandas, numpy, scipy, and matplotlib. All tests were set at a 5% significance level and a 95% confidence interval. Figures were generated programmatically in a reproducible computational workflow to preserve transparency and analytic consistency.

All data were publicly available and aggregated at the state level. No individual-level records were accessed; accordingly, the study did not constitute human-subjects research and was exempt from institutional review board review. Because this is an ecological study, associations cannot be interpreted at the individual level (ecological fallacy).

## Results

Across 51 jurisdictions (50 states and the District of Columbia), state-level maternal, perinatal, socioeconomic, and structural indicators demonstrated systematic variation when examined across the five-point legislative restrictiveness scale.

### State-level patterning across legislative context

**Fig. 1:**
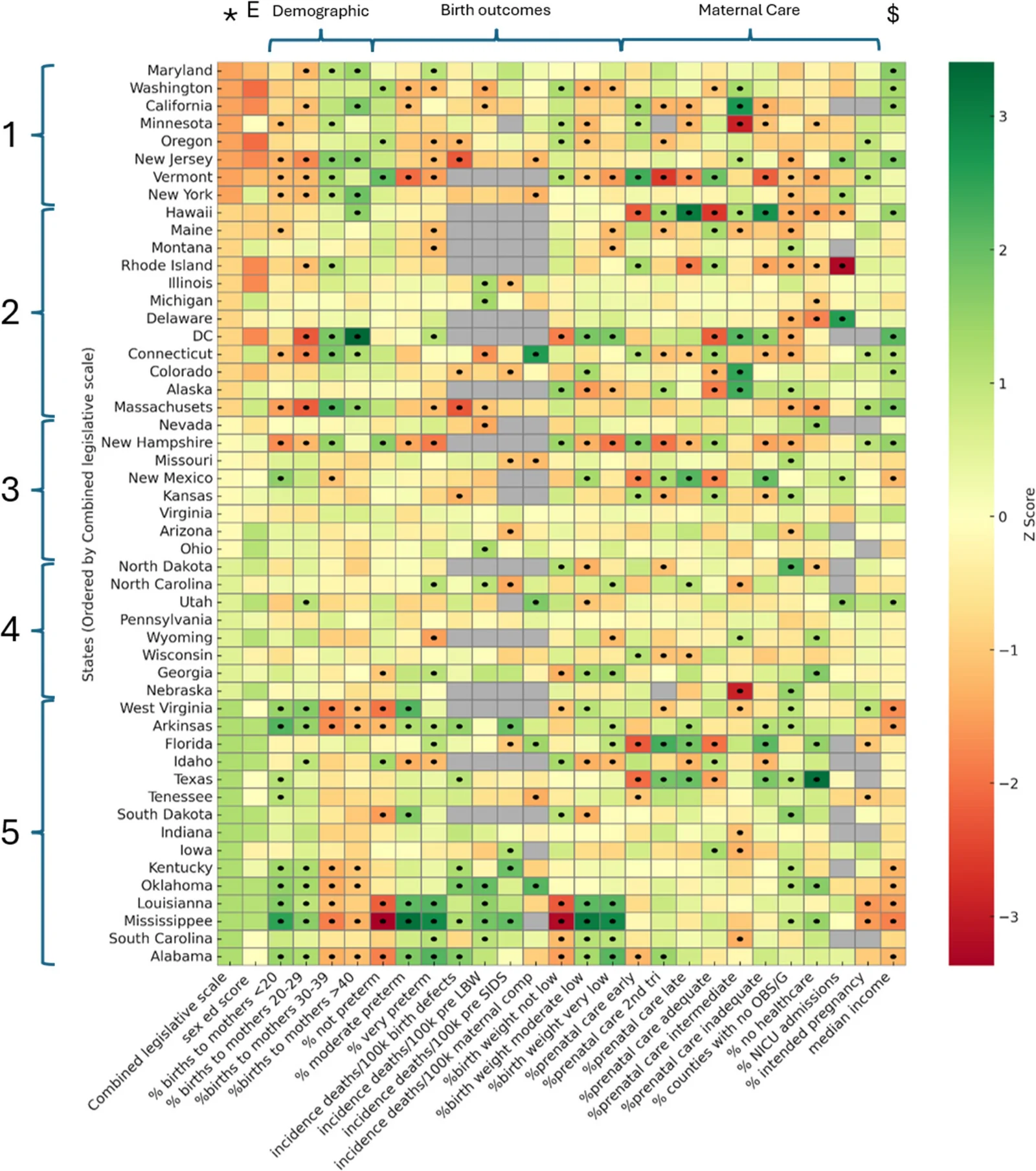
A heat map of state-level patterns in maternal age, birth outcomes, prenatal care, and socioeconomic indicators, ordered by combined legislative context. The first column (labelled at the top with *) is the combined legislative restrictiveness score ranked from 1–5 top-to bottom as indicated in the left margin; with a 1 designation for Maryland - New York, a 2 designation for Hawaii - Massachusetts, a 3 designation for Nevada - Ohio, a 4 designation for North Dakota - Nebraska, and a 5 designation for West Virginia - Alabama. Values in each column represent state-specific Z-scores color coded from green (above mean) to red (below mean) for each indicator. The Z-score was calculated by standardizing each indicator across all states. Black filled circles indicate above or below 1 standard deviation from average in each indicator. The brackets at the top show the groups of indicators; Demographic, Birth Outcomes and Maternal Care, with ($) indicating median state income, (E) indicating state SIECUS 13-scale index for sex and sexuality education, scored A + = 1 to F = 13, see methods

When indicators were standardized as Z-scores and arrayed by legislative restrictiveness (Fig. 1), a structured gradient was evident across categories. States in the least restrictive group (Categories 1–2) consistently clustered above the national mean for median household income, intended pregnancy prevalence, early-prenatal care initiation, and adequate-prenatal care. In these same jurisdictions, the proportion of births classified as not-preterm and not-low birth weight was also typically above the national average.

By contrast, states in the most restrictive category (Category 5) demonstrated positive standardized deviations for births to mothers younger than 30 years, particularly < 20 years, as well as for moderate- and very-preterm birth, moderate- and very-low birth weight, and inadequate-prenatal care. In these jurisdictions, intended pregnancy and early-prenatal care were more frequently below the national mean.

The magnitude of standard deviation was greatest at the extremes of the restrictiveness scale. Z-scores exceeding ± 1 standard deviation were concentrated in Categories 1 and 5, whereas intermediate categories (3–4) demonstrated more moderate dispersion, with most indicators falling within ± 0.5 standard deviations of the national mean. Gestational-age outcomes exhibited the clearest categorical separation, with not-preterm birth predominating in lower restrictiveness states and moderate preterm birth predominating in higher restrictiveness states.

### Monotonic associations with legislative restrictiveness

**Fig. 2:**
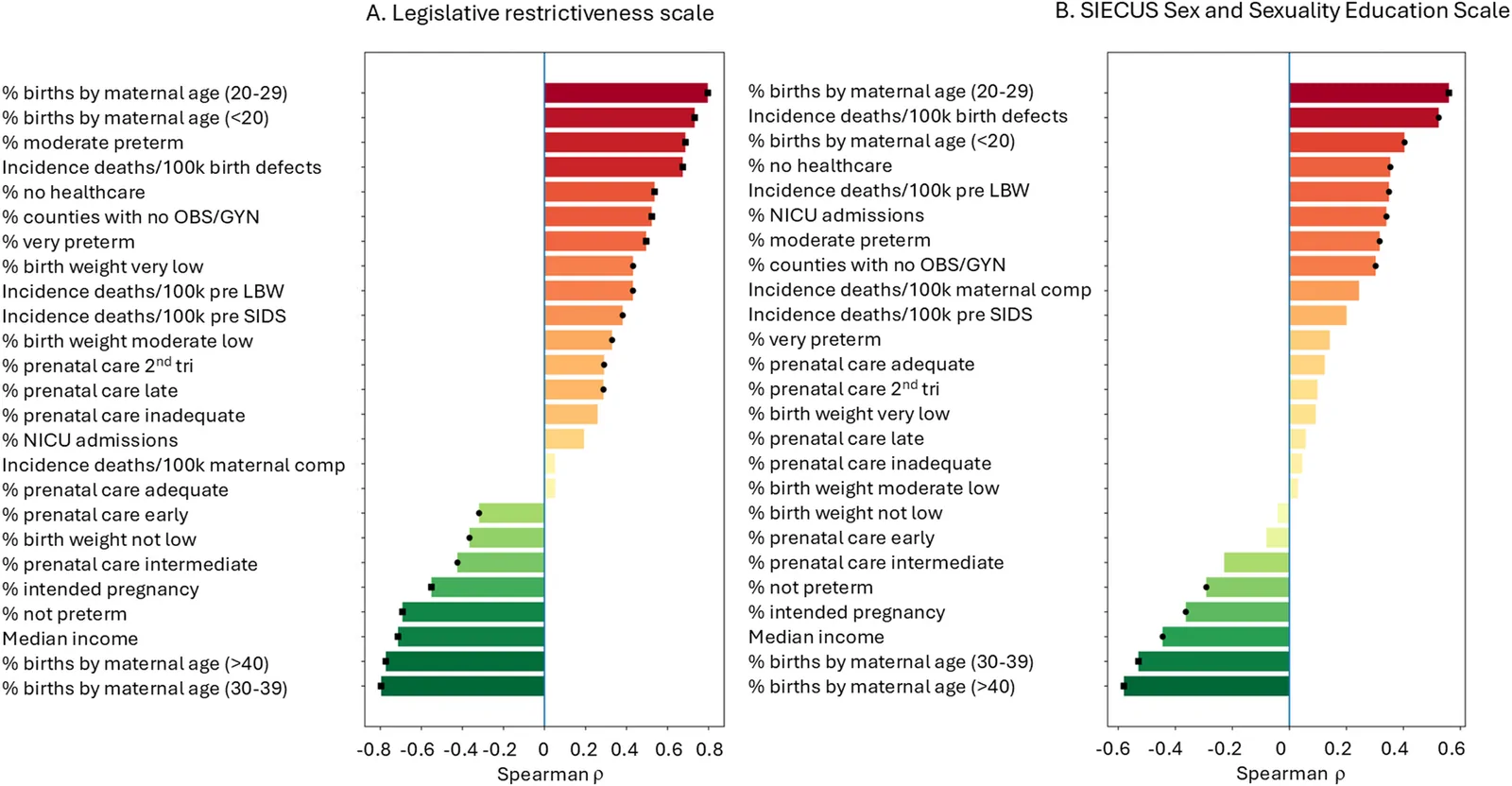
Ranked state-level correlations between legislative restrictiveness (**A**) or sex education policy (**B**), and a variety of maternal–infant health and healthcare indicators. Legislative restrictiveness scaled from 1–5 with increasing restrictiveness, SIECUS was scaled 1–13 from A + = 1 to F = 13. Correlations are ordered from highest positive to most negative within each panel. Bars are colored along a red-to-green gradient (red = positive association; green = negative association). Black circles denote *p* < 0.05 and black squares denote *p* < 0.001. **A**, Higher legislative restrictiveness was positively associated with indicators linked to younger maternal age distributions, preterm birth, low birth weight, limited obstetric access, and higher infant mortality rates. In contrast, more restrictive environments demonstrated negative associations with median income, intended pregnancy, older maternal age groups (30–39 and ≥ 40), and early or adequate prenatal care. **B** the sex education score exhibited an inverse pattern relative to restrictiveness. Higher sex education scores were positively associated with older maternal age distributions, higher median income, intended pregnancy, and early or adequate prenatal care. Negative associations were observed with adolescent births, preterm birth, low birth weight, infant mortality measures, and lack of obstetric access

To quantify these gradients, Spearman rank correlations were calculated between legislative restrictiveness and each maternal and neonatal indicator (Fig. 2A). Increasing restrictiveness was significantly negatively correlated with intended pregnancy (ρ = −0.55, *p* < 0.001) and with the proportion of births classified as not-preterm (ρ = −0.69, *p* < 0.001), representing one of the strongest correlations observed.

Conversely, legislative restrictiveness was significantly positively correlated with moderate-preterm birth (ρ = 0.69, *p* < 0.001), reflecting proportional redistribution across gestational-age categories along the restrictiveness continuum. As a comparison the same outcome indicators were correlated against the 13-point SIECUS Sex and Sexuality Education score for each state and the rank order of correlation for the outcomes had a good degree of alignment with the state legislative restrictiveness.

**Fig. 3:**
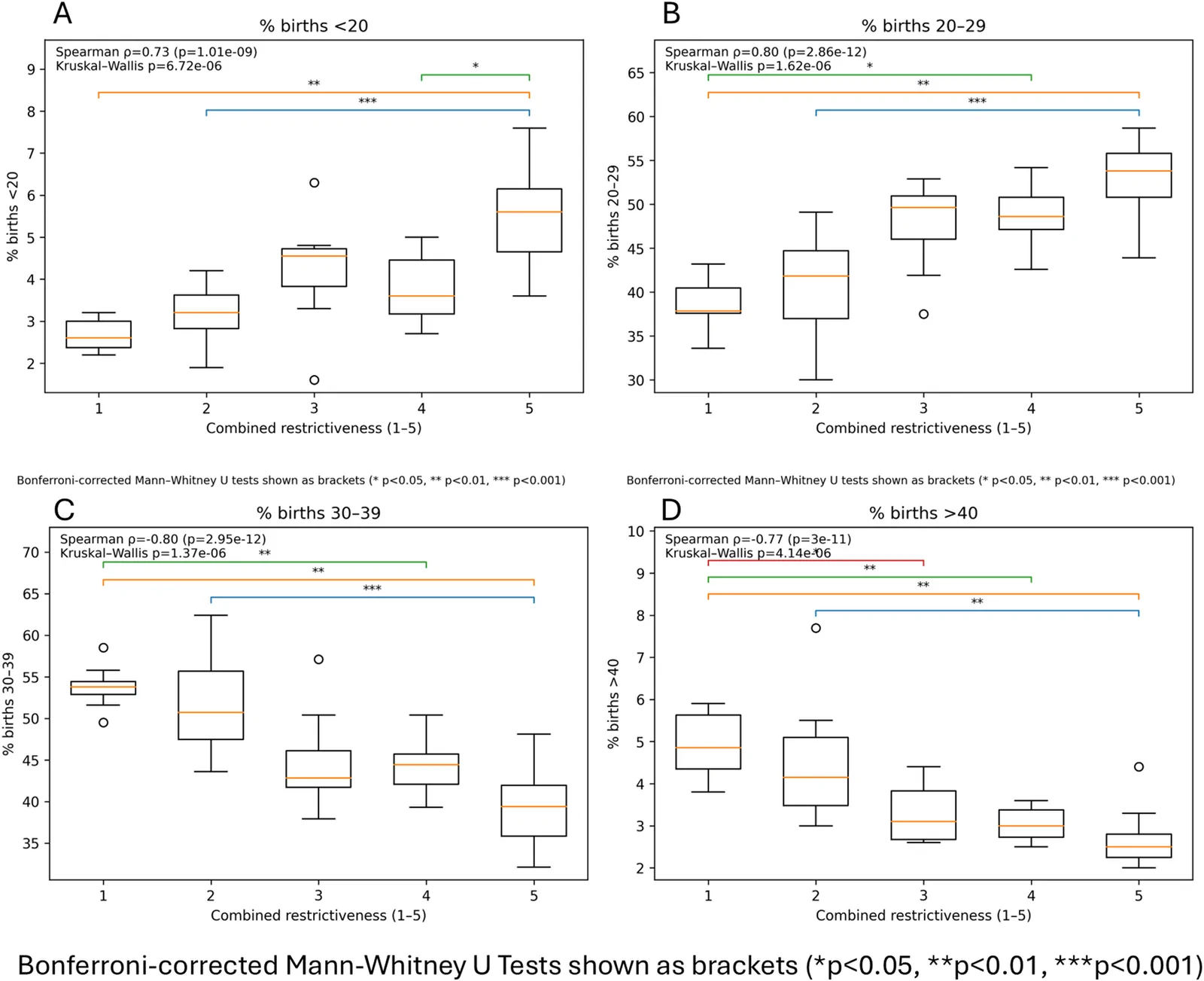
Maternal age distribution across legislative restrictiveness categories. Four-panel boxplots illustrate the association between combined legislative restrictiveness (1 = least restrictive; 5 = most restrictive) and the distribution of births by maternal age category across U.S. states. Panels display the percentage of births to mothers aged: (**A**) < 20 years, (**B**) 20–29 years, (**C**) 30–39 years, and (**D**) > 40 years. Boxplots show medians (horizontal lines), interquartile ranges (boxes), and whiskers extending to 1.5× the interquartile range. Restrictiveness is treated as an ordinal exposure variable. Spearman rank correlation coefficients (ρ) and corresponding p-values quantify monotonic associations between restrictiveness and each age category. Significant positive correlations are observed for births to mothers < 20 years and 20–29 years, indicating increasing proportions with greater restrictiveness. Conversely, significant negative correlations are observed for births to mothers aged 30–39 and > 40 years, indicating decreasing proportions across increasing restrictiveness. Overall group differences across the five restrictiveness categories were assessed using Kruskal–Wallis tests. Bonferroni-corrected pairwise Mann–Whitney U tests are shown with bracket annotations (*p < 0.05, **p < 0.01, ***p < 0.001), identifying specific between-category contrasts. Significant comparisons most consistently occur between lower (1–2) and higher (4–5) restrictiveness categories, demonstrating a graded shift in maternal age distribution

Maternal age structure also varied monotonically across the restrictiveness scale (Figs. 2A and 3). Restrictiveness showed a strong positive association with births to mothers younger than 20 years (ρ = 0.73, *p* < 0.001) and mothers aged 20–29 years (ρ = 0.80, *p* < 0.001), and a strong negative association with births to mothers aged 30–39 years (ρ = −0.80, *p* < 0.001) and mothers aged 40 years or older (ρ = −0.77, *p* < 0.001).

**Fig. 4:**
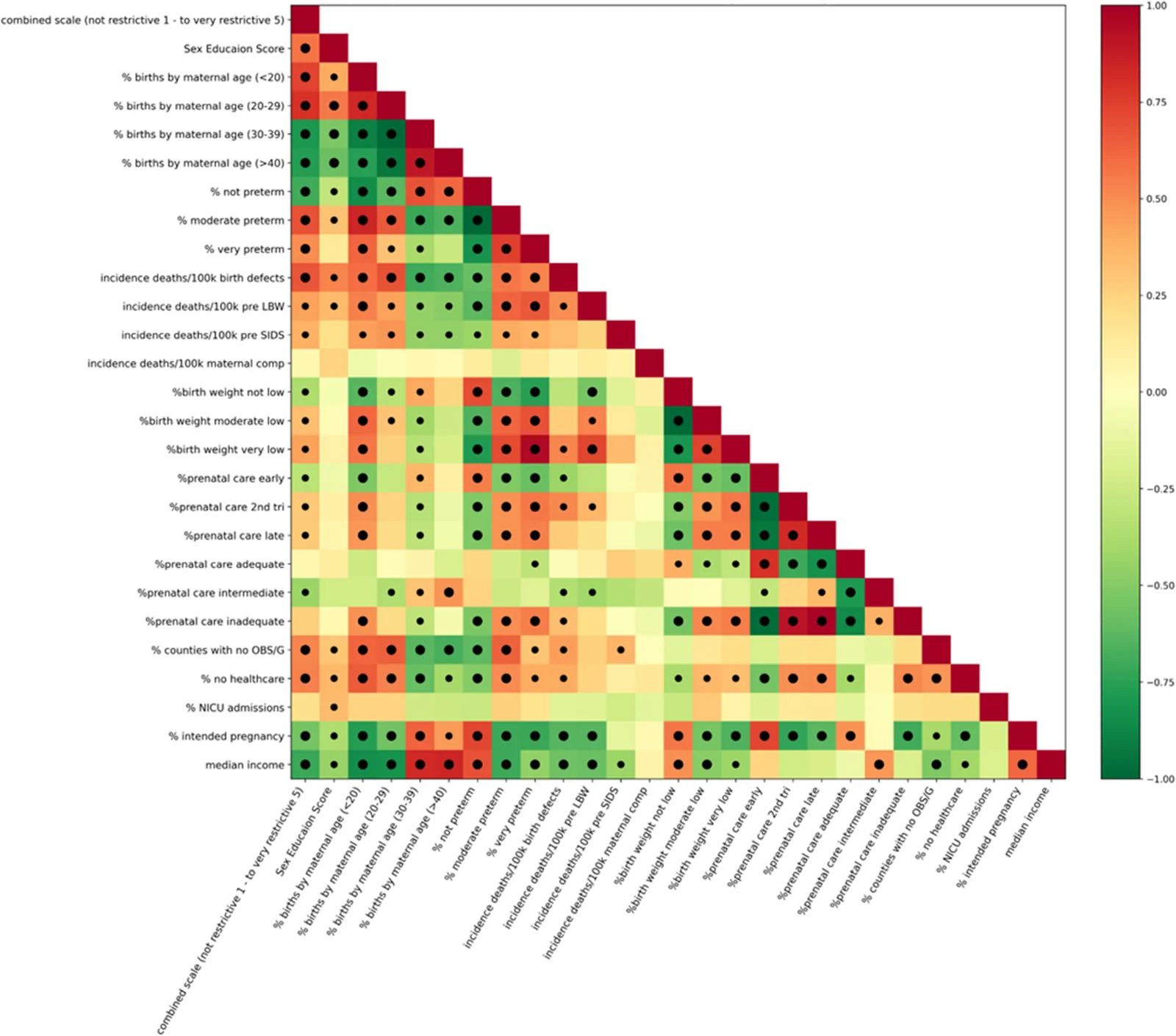
Spearman correlation matrix of maternal age, perinatal outcomes, prenatal care, and socioeconomic indicators. Spearman rank correlation matrix illustrating monotonic associations among maternal age distributions, birth outcomes, prenatal care utilization measures, health system access indicators, and socioeconomic characteristics across U.S. states. Correlation coefficients (ρ) range from − 1 to + 1 and are represented using a red-to-green gradient (red = positive association; green = negative association). Statistical significance is indicated by small black circles (*p* < 0.05) and large black circles (*p* < 0.001). Only the lower triangle of the matrix is displayed to avoid redundancy

Prenatal care timing demonstrated smaller but statistically significant associations. Early prenatal care initiation declined with increasing restrictiveness (ρ = -0.319, *p* < 0.05), whereas inadequate-prenatal care increased (ρ = 0.259, *p* < 0.05). Birth weight outcomes followed parallel directional patterns, whereby moderate and very low birth weight were positively associated with restrictiveness (ρ = 0.330 and ρ = 0.432 respectively, both *p* < 0.05), and not low birth weight was negatively associated (ρ = -0.365, *p* < 0.05, Figs. 2A and 4).

### Interrelationships among maternal age, pregnancy intention, prenatal care, and neonatal outcomes

Because higher legislative restrictiveness was associated with a greater proportion of births to mothers younger than 20 years, we examined whether state-level maternal-age distributions were also associated with pregnancy intention, prenatal-care timing, and neonatal outcomes; Fig. 4 shows the full correlation matrix for all factors. This secondary analysis was intended to characterize whether the maternal-age pattern observed across restrictiveness levels formed part of a broader cluster of state-level maternal and perinatal characteristics; it was not designed to test mediation or establish a causal pathway. The resulting correlation matrix demonstrated substantial internal coherence.

**Fig. 5:**
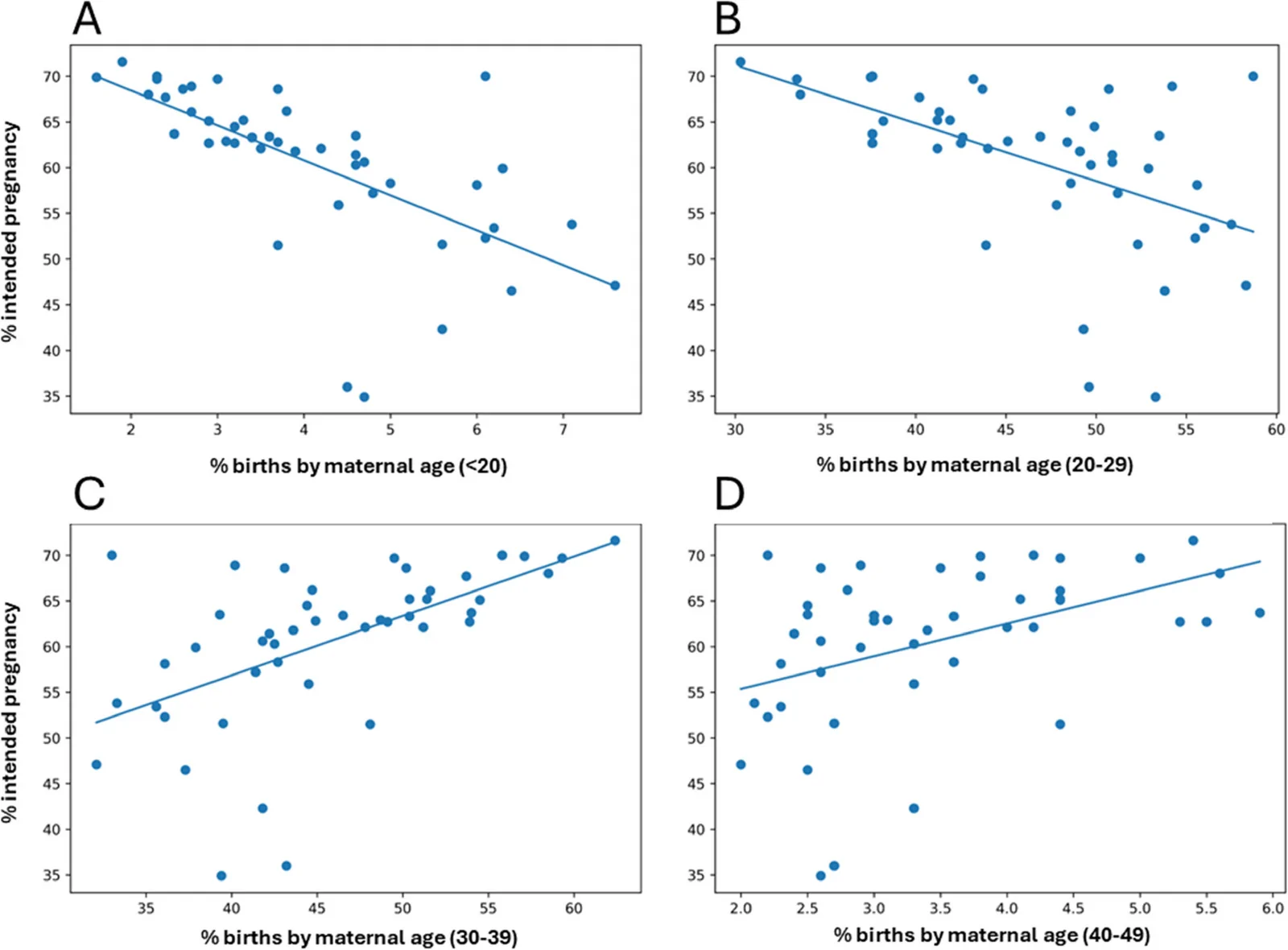
Maternal age distribution and intended pregnancy: state-level spearman association. State-level associations between maternal age distribution and the percentage of intended pregnancies across U.S. states. Each panel presents the relationship between the proportion of births within a specific maternal age group and the percentage of pregnancies reported as intended. Spearman’s rank correlation coefficient (ρ) is reported in each panel to quantify monotonic associations. Linear regression lines are shown for visual reference. **A** Mothers < 20 Years States with higher proportions of births to mothers under 20 years demonstrate a strong negative monotonic association with intended pregnancy (ρ = −0.77, *p* < 0.001). This indicates that higher adolescent birth prevalence is strongly associated with lower proportions of intended pregnancies. **B** Mothers 20–29 Years, a moderate negative association is observed between births to mothers aged 20–29 years and intended pregnancy (ρ = −0.56, *p* < 0.001), suggesting that states with larger proportions of younger adult mothers also tend to report lower intended pregnancy rates. **C** Mothers 30–39 Years In contrast, births to mothers aged 30–39 years show a strong positive association with intended pregnancy (ρ = 0.63, *p* < 0.001). States with older maternal age structures in this range tend to report higher proportions of intended pregnancies. **D** Mothers ≥ 40 Years A positive association is also observed for mothers aged ≥ 40 years (ρ = 0.46, *p* = 0.001), though of more moderate magnitude. Higher representation of this age group is associated with increased intended pregnancy prevalence

Births to mothers younger than 20 years were strongly negatively correlated with intended pregnancy (ρ = −0.77, *p* < 0.001), representing the largest single correlation observed (Fig. 5A). This age group was also negatively correlated with early prenatal care initiation (*p* < 0.001) and positively correlated with moderate-low and very-low preterm birth, moderate-low birth weight, and very-low birth weight (all *p* < 0.001) (Fig. 4).

Births to mothers aged 20–29 years demonstrated similar directional relationships, though of smaller magnitude, including a negative correlation with intended pregnancy (ρ = −0.56, *p* < 0.001), (Fig. 5B).

In contrast, births to mothers aged 30–39 years were positively correlated with intended pregnancy (ρ = 0.63, *p* < 0.001) and early prenatal care (*p* < 0.05), (Fig. 4). This age group was positively associated with not-low birth weight (ρ = 0.41, *p* = 0.003) and negatively associated with moderate-low birth weight (ρ = −0.41, *p* = 0.003) and very-low birth weight (ρ = −0.33, *p* = 0.018), (Fig. 4). Correlation magnitudes across the matrix ranged from moderate to strong (|ρ| ≈ 0.30–0.77).

### Maternal age and intended pregnancy

Given the strength of associations within the matrix, maternal age and intended pregnancy were examined directly (Fig. 5). Births to mothers younger than 20 years were strongly negatively correlated with intended pregnancy (ρ = −0.77, *p* < 0.001). Births to mothers aged 20–29 years were also negatively correlated (ρ = −0.56, *p* < 0.001).

Conversely, births to mothers aged 30–39 years demonstrated a strong positive correlation with intended pregnancy (ρ = 0.63, *p* < 0.001), and births to mothers aged 40 years or older demonstrated a moderate positive association (ρ = 0.46, *p* = 0.001). All age strata were statistically significant.

### Between-category differences in intended pregnancy

**Fig. 6:**
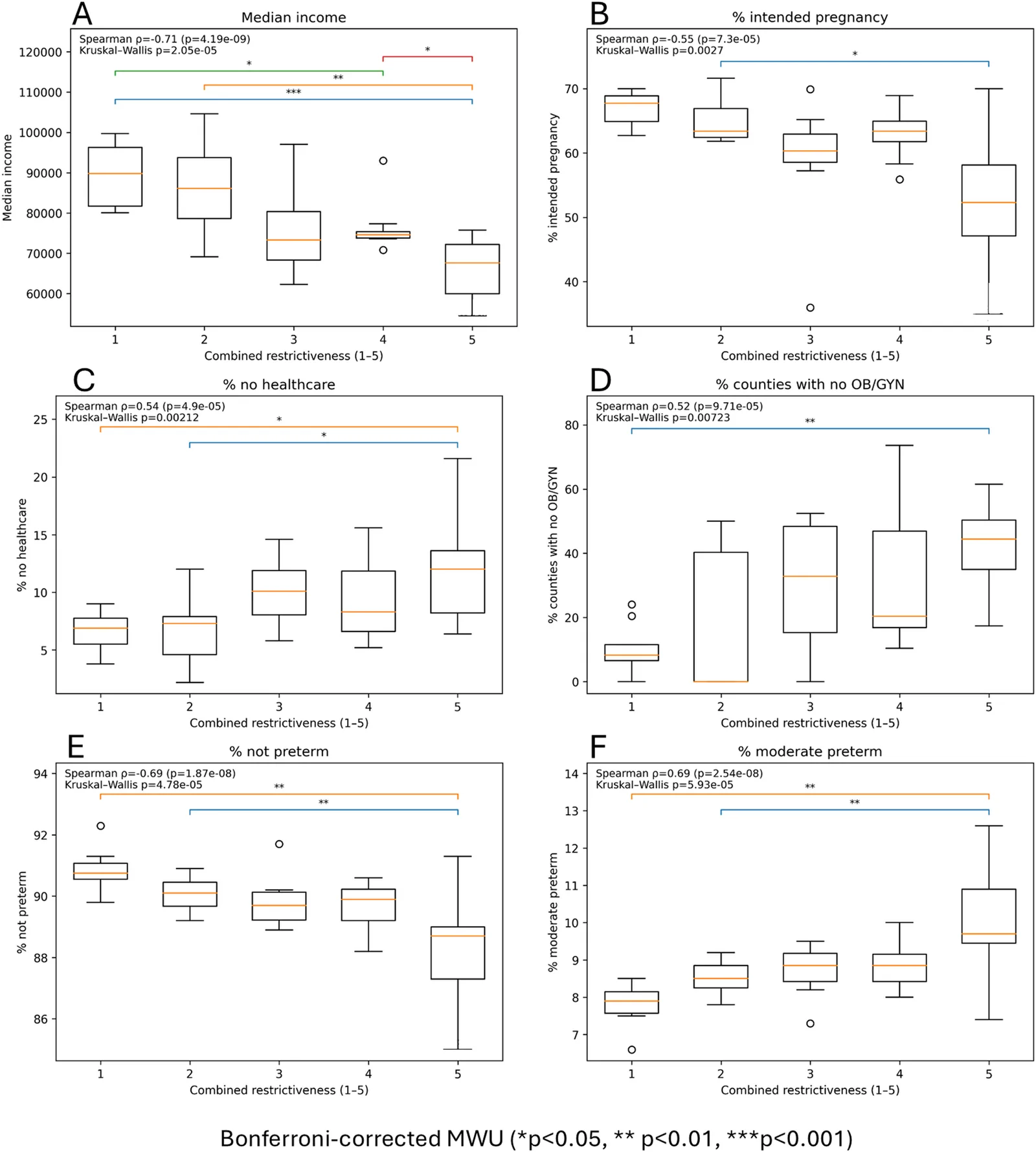
Legislative restrictiveness and state-level socioeconomic, health access, and birth outcome indicators. Six-panel boxplots illustrating associations between the combined legislative restrictiveness scale (1 = least restrictive; 5 = most restrictive) and selected state-level indicators across the United States. Panels (top to bottom, left to right) display: (**A**) Median income, (**B**) % intended pregnancy, (**C**) % population without healthcare coverage, (**D**) % counties without an OB/GYN, (**E**) % non-preterm births, and (**F**) % moderate preterm births. Boxplots represent the distribution of each indicator within restrictiveness categories (1–5). Central lines indicate medians; boxes represent interquartile ranges; whiskers extend to 1.5× IQR. Spearman rank correlations (ρ) and corresponding p-values quantify monotonic associations between restrictiveness and each indicator. Overall group differences were assessed using Kruskal–Wallis tests. Bonferroni-corrected pairwise Mann–Whitney U tests are shown with bracket annotations (*p < 0.05, **p < 0.01, ***p < 0.001). Higher legislative restrictiveness is associated with lower median income, lower intended pregnancy, higher proportions without healthcare coverage, greater county-level absence of OB/GYN services, lower proportions of non-preterm births, and higher moderate preterm birth rates, demonstrating a consistent gradient across socioeconomic context, structural access, and perinatal outcomes

When intended pregnancy was compared across restrictiveness categories (Fig. 6B), Kruskal–Wallis testing indicated statistically significant between-group differences (*p* = 0.003). Post hoc Mann–Whitney U testing with Bonferroni adjustment (α = 0.005) identified a statistically significant difference between Category 2 and Category 5 states. No other pairwise comparisons remained statistically significant after correction.

### Legislative restrictiveness and gestational-age distribution

Gestational-age outcomes demonstrated the most pronounced associations with legislative restrictiveness. When examined continuously using Spearman rank correlation (Fig. 6E-F), increasing restrictiveness was strongly negatively correlated with the proportion of births classified as not-preterm (ρ = −0.69, *p* < 0.001) and strongly positively correlated with moderate-preterm birth (ρ = 0.69, *p* < 0.001). The symmetry in magnitude reflects a proportional redistribution across gestational-age categories along the restrictiveness gradient.

Very-preterm birth demonstrated a positive correlation with restrictiveness of smaller magnitude (*p* < 0.05), indicating that the observed shift was driven primarily by changes in the moderate preterm category.

When restrictiveness was examined categorically, median proportions of not-preterm birth declined progressively from Category 1 through Category 5, whereas median proportions of moderate-preterm birth increased across the same gradient. The dispersion of values widened at higher restrictiveness levels, with Category 5 states demonstrating greater interquartile range compared with Categories 1–2.

Kruskal–Wallis testing confirmed statistically significant between-group differences for both not-preterm birth and moderate-preterm birth (*p* < 0.001 for each). Post hoc Mann–Whitney U testing with Bonferroni adjustment identified statistically significant differences between Categories 1 and 5 and between Categories 2 and 5 for both outcomes. No other pairwise comparisons remained statistically significant after correction.

The magnitude of association between restrictiveness and gestational-age distribution (|ρ| = 0.69) exceeded that observed for intended pregnancy (|ρ| = 0.55) and for most birth weight measures, indicating that gestational-age variation represented the strongest continuous correlate of legislative restrictiveness among perinatal indicators examined.

Across the restrictiveness scale, the combined pattern reflected a reduction in term births accompanied by a corresponding increase in moderate preterm births as restrictiveness increased. The association persisted when examined independently of maternal age distribution in the correlation matrix (Fig. 4), where moderate preterm birth also demonstrated significant positive correlations with adolescent maternal proportion and negative correlations with intended pregnancy.

### Maternal age and prenatal care timing

**Fig. 7:**
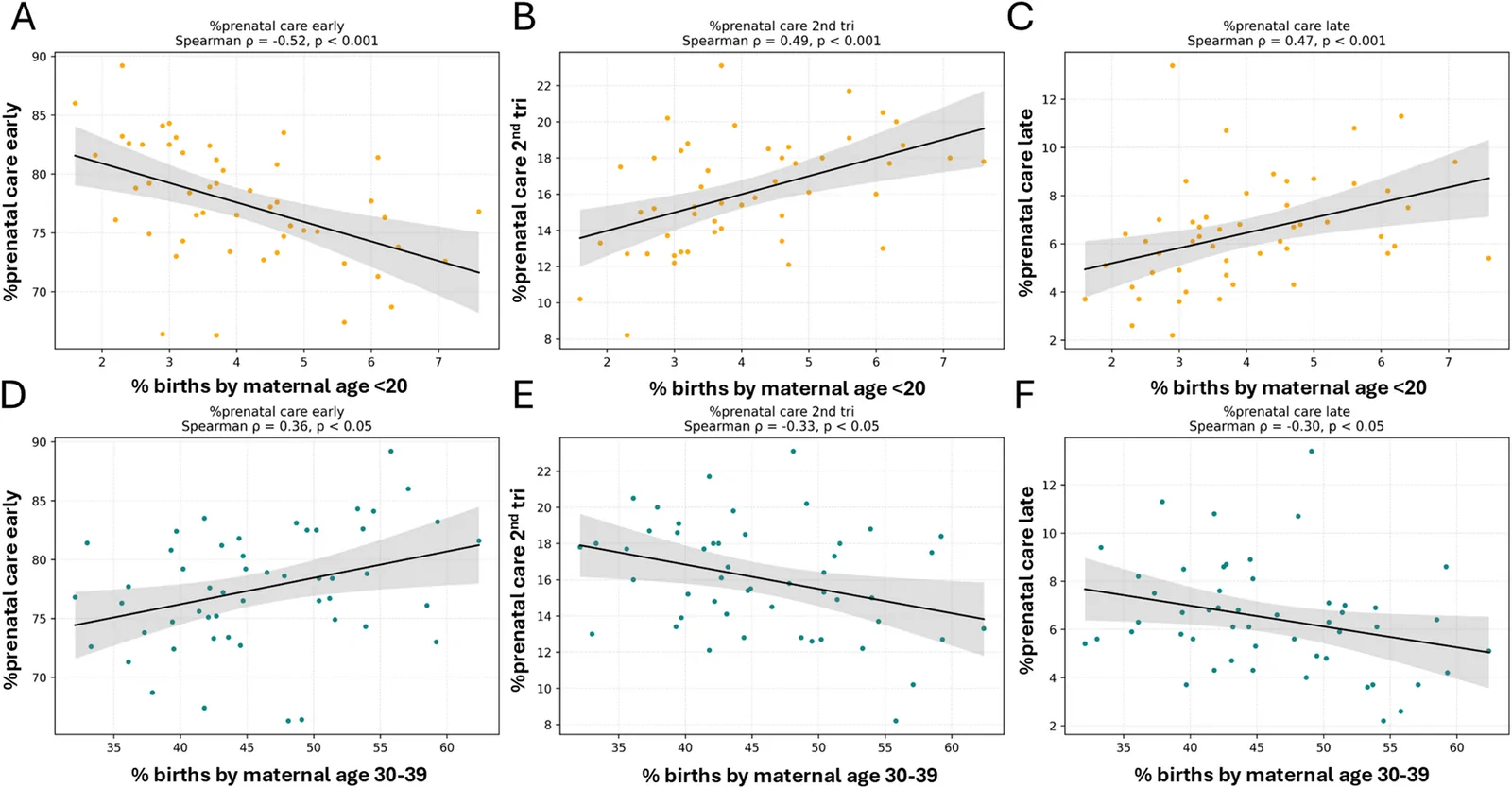
Maternal age distribution and timing of prenatal care across U.S. States. State-level associations between maternal age distribution and timing of prenatal care initiation. The top row (**A**–**C**) presents associations for births to mothers < 20 years of age, and the bottom row (**D**–**F**) presents associations for births to mothers aged 30–39 years. Each column corresponds to a prenatal care timing category: early initiation (first trimester), initiation in the second trimester, and late initiation. Among states with higher proportions of births to mothers under 20 years (**A**–**C**), a significant negative association was observed with early prenatal care (Spearman ρ = −0.52, *p* < 0.001), alongside significant positive associations with second-trimester care (ρ = 0.49, *p* < 0.001) and late care (ρ = 0.47, *p* < 0.001). In contrast, states with higher proportions of births to mothers aged 30–39 years (**D**–**F**) exhibited the inverse pattern. Positive associations were observed with early prenatal care (ρ = 0.36, *p* < 0.05), while negative associations were observed with second-trimester (ρ = −0.33, *p* < 0.05) and late prenatal care (ρ = −0.30, *p* < 0.05). Trendlines with 95% confidence intervals are shown for visualization, and Spearman’s rank correlation coefficients quantify monotonic associations

We next examined maternal age in relation to timing of prenatal care initiation (Fig. 7). Higher proportions of births to mothers younger than 20 years were negatively correlated with early prenatal care (ρ = −0.52, *p* < 0.001) and positively correlated with second-trimester (ρ = 0.49, *p* < 0.001) and late initiation (ρ = 0.47, *p* < 0.001). Births to mothers aged 30–39 years were positively correlated with early prenatal care (ρ = 0.36, *p* < 0.05) and negatively correlated with delayed initiation (*p* < 0.05), (Fig. 4).

### Maternal age and birth weight

**Fig. 8:**
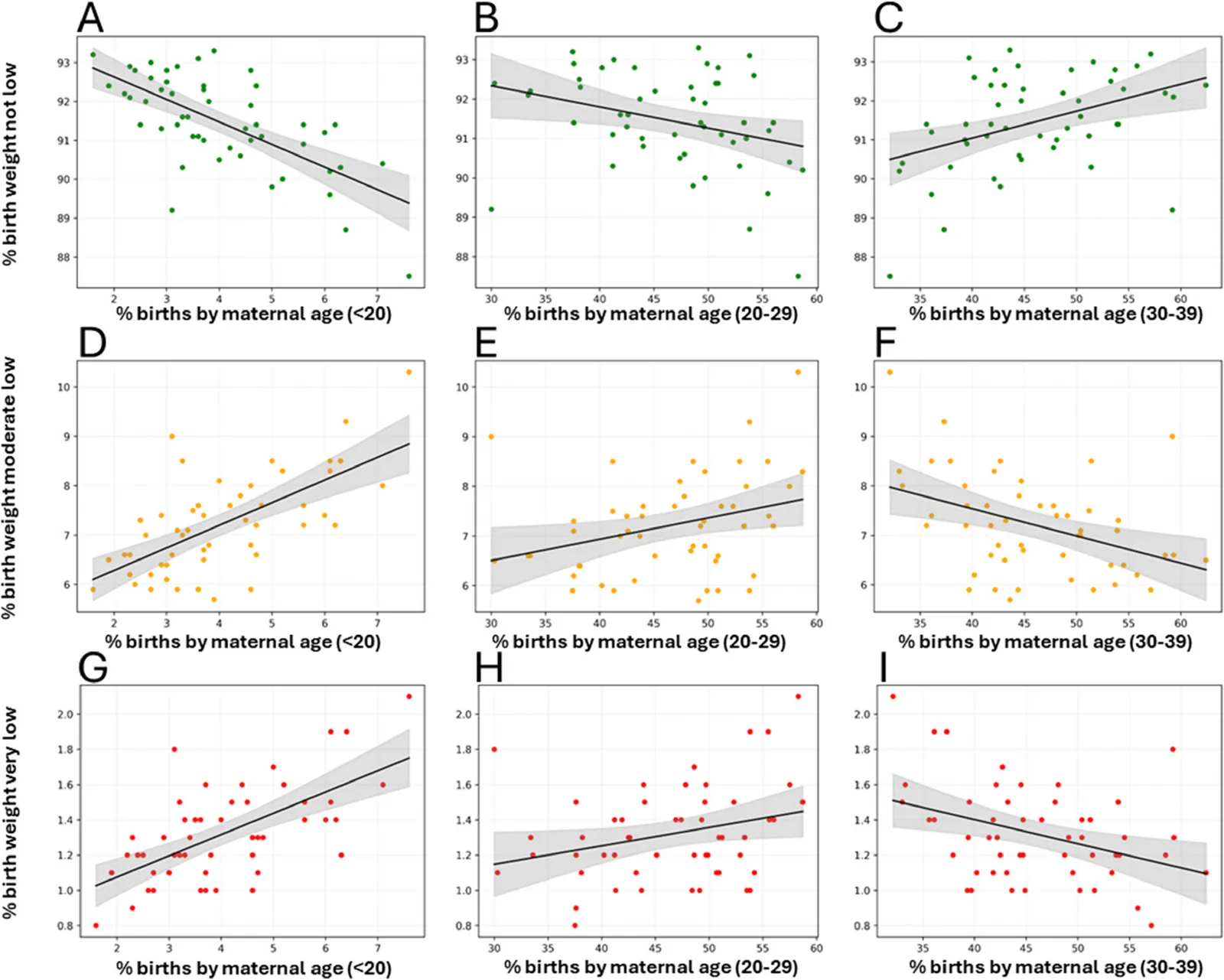
Maternal age distribution and birth weight outcomes across U.S. States. State-level associations between maternal age distribution and birth weight outcomes. Columns correspond to maternal age categories (< 20 years, 20–29 years, and 30–39 years), and rows correspond to three birth weight outcomes: percentage of births not low birth weight (top row), percentage of moderate low birth weight (middle row), and percentage of very low birth weight (bottom row). Spearman’s rank correlation coefficients (ρ) were calculated and linear trendlines with 95% confidence intervals are included for visualization. For births to mothers < 20 years (**A**, **D**, **G**), strong and consistent associations are observed. Higher proportions of adolescent births are associated with lower percentages of not low birth weight (ρ = −0.63, *p* < 0.001) and higher percentages of moderate (ρ = 0.61, *p* < 0.001) and very low birth weight (ρ = 0.57, *p* < 0.001). For mothers aged 20–29 years (**B**, **E**, **H**), associations are directionally similar but more moderate. The proportion of births in this age group is negatively associated with not low birth weight (ρ=−0.31, *p* = 0.024) and positively associated with moderate low birth weight (ρ = 0.31, *p* = 0.026), while the association with very low birth weight is positive but not statistically significant (ρ = 0.26, *p* = 0.066). In contrast, higher proportions of births to mothers aged 30–39 years (**C**, **F**, **I**) demonstrate the inverse pattern. This age group is positively associated with not low birth weight (ρ = 0.41, *p* = 0.003) and negatively associated with moderate (ρ = −0.41, *p* = 0.003) and very low birth weight (ρ=−0.33, *p* = 0.018)

Finally, birth weight distributions were examined by maternal age strata (Fig. 8). For births to mothers younger than 20 years, not-low birth weight was negatively correlated (ρ = −0.63, *p* < 0.001), whereas moderate-low birth weight (ρ = 0.61, *p* < 0.001) and very-low birth weight (ρ = 0.57, *p* < 0.001) were positively correlated.

Births to mothers aged 20–29 years demonstrated weak statistically significant associations with moderate-low birth weight (ρ = 0.31, *p* = 0.026) and not-low birth weight (ρ = −0.31, *p* = 0.024).

Births to mothers aged 30–39 years were positively correlated with not-low birth weight (ρ = 0.41, *p* = 0.003) and negatively correlated with moderate-low birth weight (ρ = −0.41, *p* = 0.003) and very-low birth weight (ρ = −0.33, *p* = 0.018).

### Summary of observed associations

Across sequential analyses, increasing legislative restrictiveness was significantly associated with higher proportions of births to mothers younger than 30 years, lower intended pregnancy prevalence, reduced early prenatal care initiation, higher moderate preterm birth, and higher moderate and very low birth weight. Observed effect sizes ranged from moderate to strong (|ρ| ≈ 0.30–0.77). All findings derive from cross-sectional ecological state-level analyses.

## Discussion

This ecological analysis identified a consistent gradient linking more restrictive state abortion-policy environments with less favorable maternal and perinatal profiles. Across jurisdictions, higher restrictiveness was associated with younger maternal age distributions, lower intended pregnancy prevalence, less early prenatal care, greater moderate preterm birth, and more low-birth-weight births. The internal coherence of these findings across related domains suggests that the observed pattern is not random noise but part of a broader population-health configuration.

The results are substantively consistent with a rapidly expanding literature documenting associations between reproductive policy environments and health outcomes beyond abortion service utilization alone. Prior studies have reported associations between restrictive abortion policy and higher maternal mortality, adverse obstetric outcomes, and infant mortality [6–9, 12–14], while newer post-*Dobbs* studies have documented changes in infant survival and maternal morbidity during periods following the implementation of state bans [10, 11]. The present study adds to that literature in two ways. First, it evaluates a wider bundle of outcomes, including pregnancy intention, prenatal engagement, maternal age structure, gestational age, birth weight, and structural access, in a single comparative framework. Second, it interprets abortion-policy restrictiveness not only as a legal exposure but also as a proxy for a broader reproductive-health ecosystem that may include differences in coverage, geography of care, public-health investment, and educational climate.

### Legislative restrictiveness and the structural gradient of risk

The strongest continuous association in the present analysis involved gestational-age distribution, with lower proportions of term birth and higher proportions of moderate preterm birth observed at higher restrictiveness levels. Gestational age is associated with multiple co-occurring factors, including pregnancy intention, maternal health status, stress, access to timely care, and continuity of obstetric services; however, the present design cannot determine which factors account for the observed pattern. Prior studies of restrictive abortion policy and adverse birth outcomes have found similar relationships for preterm birth and low birth weight, although effect sizes and specific policy measures vary across designs [8, 9, 12].

Heterogeneity within the most restrictive states is also important. Not every restrictive state performed equally poorly across indicators, which suggests that abortion policy does not operate in isolation. Some states may partly buffer risk through stronger clinical infrastructure, broader insurance coverage, or other maternal-health supports, while others may combine restrictive legal environments with deeper structural deficits. This interpretation is consistent with March of Dimes evidence that maternity care access, uninsured prevalence, and travel burden vary substantially across states and counties [19, 20].

Accordingly, abortion-policy restrictiveness should be interpreted here as part of a structural gradient of risk rather than as a single deterministic cause. That distinction matters for interpretation as maternity-care capacity, public insurance design, and preventive reproductive services may covary with both abortion-policy restrictiveness and maternal and neonatal outcomes.

### Maternal age, pregnancy intention, and perinatal risk

Maternal age distribution is relevant to interpreting the primary policy analysis because higher proportions of births to mothers younger than 20 years were observed in more restrictive states and were also associated with lower intended pregnancy, less early prenatal care, and less favorable gestational-age and birth-weight profiles. Figure 4 therefore places the association between policy restrictiveness and maternal age within the wider pattern of state-level reproductive and perinatal indicators. These ecological correlations do not establish that maternal age mediates or explains the associations between policy context and the measured outcomes, but they demonstrate why maternal age is an important contextual variable. The observed relationships are also consistent with prior evidence linking unintended pregnancy to poorer maternal-infant outcomes and lower antenatal care use [15, 16].

The sex education context may be associated with part of this pattern. A large review of school-based comprehensive sexuality education found consistent benefits for sexual health knowledge and related behaviors, and quasi-experimental evidence suggests that more comprehensive sex education reduces teen births at the population level [17, 18]. In that sense, the maternal age gradient observed here may reflect not only care access during pregnancy but also upstream differences in reproductive planning before pregnancy occurs.

National data show that teen birth rates continued to decline through 2023, which is important context for interpreting the present state-level results [21]. The ecological finding here is not that adolescent childbearing is increasing nationally, but rather that its relative concentration remains greater in more restrictive policy environments, where it appears to cluster with lower intended pregnancy and less favorable perinatal outcomes.

### Prenatal care timing and engagement

The prenatal-care findings were statistically weaker than the gestational-age findings, but they remain meaningful. Early prenatal care was lower and inadequate care higher in more restrictive settings, while younger maternal age was associated with later prenatal care initiation. This is consistent with the literature showing that pregnancy intention, affordability, and health system access influence when antenatal care begins [15, 16].

At the same time, prenatal care timing is an imperfect proxy for care quality. A state may show similar first-trimester entry rates while differing substantially in care continuity, specialty access, insurance coverage, or emergency obstetric capacity. This may help explain why the gestational-age and birth-weight gradients were more pronounced than the prenatal-timing gradients in the present analysis.

More broadly, maternity care access in the United States is uneven. March of Dimes reported in 2024 that more than one-third of U.S. counties qualified as maternity care deserts and that living in a maternity care desert was associated with increased preterm birth risk [20]. These access gaps may be part of the broader structural context accompanying the observed geographic patterning of maternal and neonatal outcomes; however, this ecological analysis cannot determine whether they mediate the association with policy restrictiveness.

### Sex education and other structural factors

The clustering of abortion policy, socioeconomic context, and sex education measures in this study suggests that reproductive legislation should not be understood narrowly as a single-issue policy domain. Rather, it appears embedded in a larger package of state decisions about public investment, health coverage, education, and family support. Other research has likewise shown that the consequences of restricted abortion access can extend beyond clinical episodes of care to include child welfare systems [24].

This broader framing suggests that abortion-policy restrictiveness may function as an indicator of a wider state context. The present analysis establishes that restrictiveness co-occurs with differences in reproductive autonomy, sex and sexuality education, preventive health infrastructure, and social supports, but it cannot determine causal pathways among these factors.

### Limitations

Legal uncertainty, interstate travel, and delays in obtaining reproductive care may be important features of the post-*Dobbs* policy environment; however, none of these factors were measured in the present study. Consequently, this analysis cannot determine whether they are associated with the observed state-level patterns in pregnancy intention, maternal age distribution, prenatal-care timing, or perinatal outcomes.

Future longitudinal and multilevel studies should directly examine the timing and stability of policy changes alongside measures of patient travel, delays in care, and patient and clinician experiences of legal uncertainty. Guttmacher estimates indicate that interstate travel for abortion care remained substantially above the pre-*Dobbs* baseline in 2023 and 2024, and prior research has documented financial and social burdens associated with such travel [5, 25]. Incorporating these measures into future studies could clarify their relationships with maternal and neonatal outcomes.

Several limitations warrant cautious interpretation of the findings. First, this was a cross-sectional ecological study; therefore, the analysis cannot support individual-level inference or establish causality. The observed associations may reflect residual confounding by race and ethnicity, rurality, Medicaid expansion status, clinician supply, poverty, healthcare infrastructure, or other policy and demographic characteristics that co-occur with abortion restrictions.

The harmonized restrictiveness scale also necessarily simplifies complex and changing legal environments. Although it captures the broad policy climate within each state, it cannot account for every difference in enforcement, temporary injunctions, provider behavior, local service availability, or within-state geographic variation.

There was also a temporal mismatch between the exposure and outcome data. The policy classification reflected the legal environment in 2025, whereas several outcomes drew data from 2020 to 2025 and therefore included observations predating the 2022 Dobbs decision and subsequent state-level policy changes. The associations should consequently not be interpreted as effects of these recent legal changes. They may partly reflect preexisting differences among states, and the full health consequences of newer policies may not yet be captured in the available data. Nevertheless, the consistency of associations across conceptually related indicators supports the usefulness of the framework for surveillance and hypothesis generation. Longitudinal data would have been ideal to examine health outcomes pre-and post-Dobbs but this timeline coincided with the COVID-19 pandemic and significant confounding of healthcare provision.

The analysis was limited to PeriStats outcome categories. Post-term birth (> 42 weeks) and macrosomia (> 4,000 g) were not analyzed separately, so the “not-preterm” and “not-low-birth-weight” categories may include births with these adverse outcomes. The findings may therefore not capture other clinically important dimensions of maternal and neonatal health.

## Conclusion

The findings support treating reproductive legislation as crucial to public health policy. Even without causal claims, the pattern observed here suggests that states with more restrictive abortion-policy environments may face compounded risk across pregnancy intention, prenatal engagement, gestational age, and birth weight. Legal clarity and clinically workable rules matter. Rapidly changing or ambiguous reproductive laws may coincide with differences in access, travel burden, and uncertainty for patients and providers [3, 4]. Upstream prevention matters. Evidence-based comprehensive sexuality education is associated with lower teen birth rates and better reproductive planning, making it a plausible lever for improving maternal age structure and pregnancy intention at the population level [17, 18]. Maternal health infrastructure matters. Expanding obstetric access, reducing maternity care deserts, and addressing insurance gaps are likely necessary complements to any reproductive policy strategy intended to improve maternal and neonatal outcomes [8, 19, 20].

In this ecological analysis, state abortion-policy restrictiveness was systematically associated with intended pregnancy, maternal age distribution, prenatal engagement, gestational age, and birth weight across the United States. The strongest observed gradients involved gestational age and pregnancy intention, with more restrictive policy environments tending to cluster with younger maternal age profiles and less favorable perinatal indicators.

These findings do not establish that abortion legislation causes worse maternal or neonatal outcomes. They do, however, support the argument that reproductive policy is embedded within the broader structural architecture of maternal health. In the post-Dobbs United States, abortion legislation should be understood not only as a legal issue, but also as a population health indicator with potential implications for geographic inequality in maternal and infant outcomes.

## Data Availability

The datasets analyzed during the current study are publicly available from the following sources. The March of Dimes PeriStats data are available at https://www.marchofdimes.org/peristats/ through the PeriStats data portal. The Guttmacher Institute policy data are available at https://states.guttmacher.org/policies/ through its interactive policy map. The Center for Reproductive Rights policy data are available at https://reproductiverights.org/maps/abortion-laws-by-state/ through its U.S. abortion laws map. The compiled dataset used for analysis is available from the corresponding author on reasonable request.

## Abbreviations

NICU: Neonatal Intensive Care Unit
IRB: Institutional Review Board
Z-score: Standardized score
OB/GYN: Obstetrician-Gynecologist
